# Polymer photonic microstructures for quantum applications and sensing

**DOI:** 10.1007/s11082-017-0922-x

**Published:** 2017-02-13

**Authors:** Sebastian Knauer, Felipe Ortiz Huerta, Martín López-García, John G. Rarity

**Affiliations:** 1 Bristol Centre for Nanoscience and Quantum Information, University of Bristol, Tyndall Avenue, Bristol, BS8 1FD UK; 2 Department of Electrical and Electronic Engineering, University of Bristol, Merchant Venturers Building, Woodland Road, Bristol, BS8 1UB UK

**Keywords:** Polymer resonator, Cavity, Colour centres, Numerical simulation

## Abstract

We present modelling results for efficient coupling of nanodiamonds containing single colour centres to polymer structures on distributed Bragg reflectors. We explain how hemispherical and super-spherical structures redirect the emission of light into small numerical apertures. Coupling efficiencies of up to 68.5% within a numerical aperture of 0.34 are found. Further, we show how Purcell factors up to 4.5 can be achieved for wavelength scale hemispheres coated with distributed Bragg reflectors. We conclude with an experimental proposal for the realisation of these structures.

## Introduction

Colour centres in nanodiamonds are widely used single photon sources, with applications including quantum information processing (Hanson and Awschalom 2008; Maurer et al. 2012), thermometry (Neumann et al. 2013), magnetometry (Taylor et al. 2008), and fluorescence bio-markers (Balasubramanian et al. 2014). The most widely studied colour centre is the nitrogen-vacancy centre, consisting of a substitutional nitrogen adjacent to a vacancy ($\hbox {NV}^{-}$ centre). It possesses a ground state spin, which can be optically initialised, manipulated with microwave radiation and optically read out. Stable nanodiamond emitters with long coherence times have been reported (Knowles et al. 2014). To increase both the coupling efficiency between $\hbox {NV}^{-}$ centres in nanodiamond, and speed of spin read-out, photonic structures are required (e.g. Barth et al. 2010; Mader et al. 2015). Laser writing in photo-polymer is a promising route for the fabrication of biocompatibile cost effective structures in high quantities. For example polymer nanobeams (Tadayon et al. 2014) and droplet whispering gallery resonators (Ta et al. 2013) have been reported. However, the deterministic coupling of nanodiamonds containing $\hbox {NV}^{-}$ centres to polymer photonic structures for fluorescence enhancement and directional coupling remains challenging (Schell 2013).

**Fig. 1:**
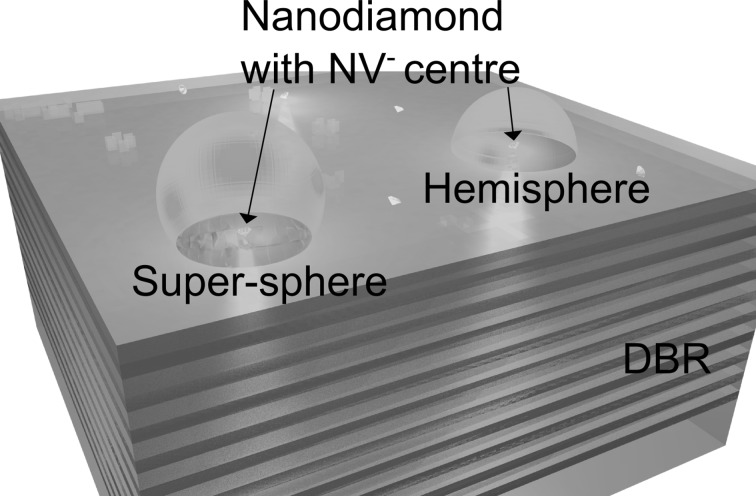
Concept of nitrogen-vacancy centres in nanodiamonds addressed to hemispheres and super-spheres on a distributed Bragg reflector

To address some of these challenges we present two types of structures on a distributed Bragg reflector (DBR) (Fig. 1) where the DBR confines and enhances the $\hbox {NV}^{-}$ centre’s emission in the upwards direction. First, we discuss two types of top polymer structures: hemispherical and super-spherical structures, which allow enhanced collection efficiencies into smaller numerical apertures than bare DBR surface emission. Then we discuss the potential for full cavity structure to enhance the interaction rate between cavity mode and $\hbox {NV}^{-}$ centre, which allows us to concentrate the emission into a narrow spectral band and to enhance emission into the $\hbox {NV}^{-}$ centre’s zero-phonon line. A commercial-grade simulator based on the finite-difference time-domain (FDTD) method was used to perform the calculations (Lumerical Solutions Inc.).

## Numerical modelling

### Distributed Bragg reflector

**Fig. 2:**
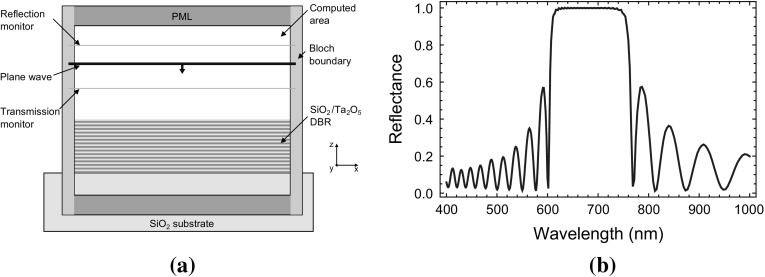
**a** Simulation setup for the analysis of the distributed Bragg reflector. **b** Simulated stop band for a 16 layer silicone dioxide/tantalum pentoxide distributed Bragg reflector. The stop band shows a reflectance of over 99.9% between 600 and 750 nm (normal incidence)

The design of the DBR has been guided by the premise of a wide stop band reaching from 600 to 750 nm with a centre wavelength of 660 nm. This band gap is designed to maintain high reflectivity at 637 nm (zero-phonon line $\hbox {NV}^{-}$ centre) out to high incidence angles ($\theta \le 40^{\circ }$). For the simulation silicon dioxide ($\mathrm {SiO_2}$) with a refractive index of $n_{\mathrm {SiO_2}}=1.54$ is used as low index quarter wavelength top layer to ensure an antinode at the mirror surface. This is then alternated with quarter wavelength tantalum pentoxide ($\mathrm {Ta_2O_5}$) layers with a refractive index of $n_{\mathrm {Ta_2O_5}}=2.03$ (Fig. 2a). To obtain the reflectance, we launch a broad band plane wave in the simulation region, measure the reflected signal and normalise it to the emitted plane wave signal itself. We use perfectly matched layers (PML) in the z-directions and Bloch boundary conditions on x/y-directions to maintain the phase. A total of sixteen pairs ensures an over 99.9% reflectance (mid band normal incidence) (Fig. 2b). Semi-analytic analysis of dielectric stack reflectivity involve use of the transfer matrix theory (Yeh et al. 1977) and these approaches were used for the case of the single emitter close to a mirror in seminal works such as (Drexhage 1974). These semi-analytic approaches can be used to model various application scenarios such as optical switches (Zhenhua et al. 2013, 2016). Here we continue to use FDTD methods as our systems consist of more complex 3D structures combined with simple dielectric stacks.

**Fig. 3:**
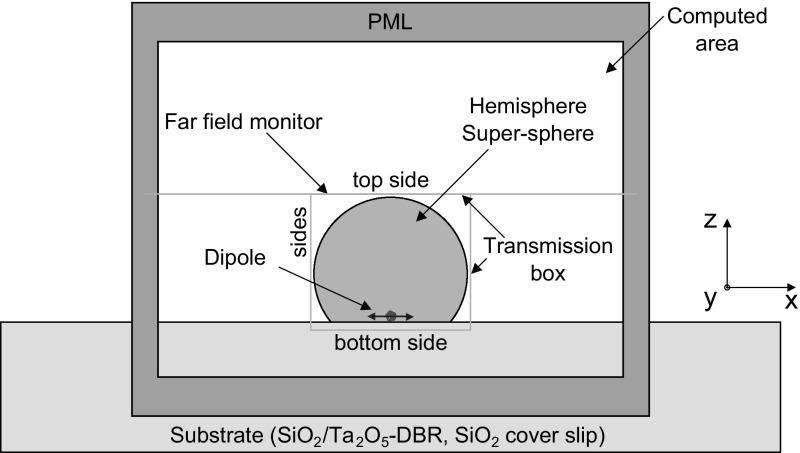
Simulation setup for an $\hbox {NV}^{-}$ centre (dipole) coupled to polymer hemispheres and super-spheres

### Polymer structures

Hemispheres, or so-called solid immersion lenses, are widely studied and used, e.g. Hadden et al. (2010). Light can be more efficiently coupled and collected, for example to or from a solid state emitter like an $\hbox {NV}^{-}$ centre. A bigger challenge in the fabrication process are super-spheres, also called Weierstraß spheres. Some freestanding examples made out of glass (Karrai et al. 2000), gallium phosphide (Wu et al. 1999), silicon (Serrels et al. 2008a) or gallium arsenide (Liu et al. 2005) have been demonstrated. In the simulations presented here, we show how single dipoles, i.e $\hbox {NV}^{-}$ centres, on the surface of the DBR can be coupled to these polymer hemispheres and super-spheres. The FDTD simulation setup is shown in Fig. 3. The dipoles with a centre wavelength of 637 nm and a pulse length of 20 fs have been placed at the position at the centre of the hemispheres/super-sphere, 30 nm above the surface of the DBR. We calculate the emitted field on top of the hemisphere/super-sphere in the near field and project it into 1 m distance to obtain the far field, as indicated in Fig. 3 by far field monitor, which allows us to calculate the angular response of the field. Furthermore, we calculate the emitted power at the outside of the hemisphere/super-sphere (transmission box) and normalise the signal to the actual dipole emitted power. Here we study the signal emitted into the entire top, covered by the top sides and the sides, and into the substrate, covered by bottom side. We take the signal through our frequency monitor in 20 nm distance from the hemisphere/super-sphere interfaces and DBR substrate respectively. Field outside the simulation region is absorbed by perfectly matched layers. Moreover, we discuss the difference between the substrate based on $\mathrm {SiO_2}$/$\mathrm {Ta_2O_5}$ distributed Bragg reflectors (DBR) and for comparison $\mathrm {SiO_2}$ cover slips. The optimization is performed for different radii ranging from 1 to $10\,\upmu \hbox {m}$.

**Fig. 4:**
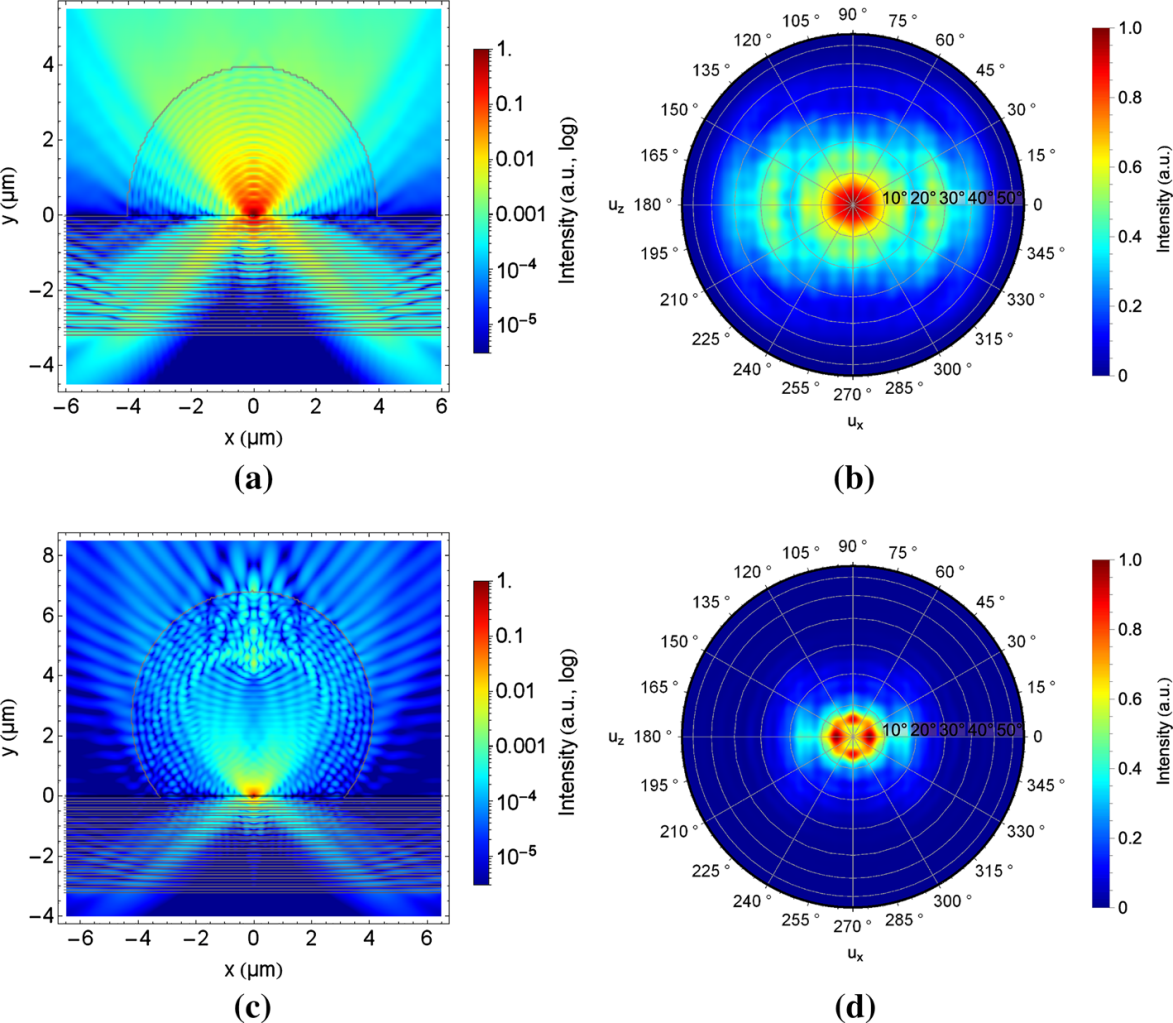
Polymer hemisphere (**a** electric field magnitude, **b** corresponding far field) and polymer super-sphere (**c** electric field magnitude, **d** corresponding far field) with radius $4.16\,\upmu \hbox {m}$ on a $\mathrm {SiO_2/ Ta_2O_5}$ DBR. The fields are calculated at 637 nm

First, we focus on the hemisphere results. An example of the optimal coupling out of these hemisphere (radius $4.16\,\upmu \hbox {m}$) on top of the DBR is shown with the electric field magnitude in Fig. 4a. The high reflectivity of the DBR at normal incidence is clearly seen contrasting with light leakage through the DBR at high incidence angles. The resulting directional guidance of the light in the vertical direction is shown in the far field projections Fig. 4a. For the DBR substrate most of the upward light is confined within a half angle of $15^{\circ }$, relating to a numerical aperture of approximately 0.5, but part of the light is emitted up to a numerical aperture of 0.76. For collection efficiency we observe 65% of the emitted light leaves through the top of the hemisphere (into $2\pi$ steradians) while 35% leaks through the DBR substrate. These values are measured at the central wavelength of 637 nm. It is observed that the fraction of light coupling through the top oscillates with different radii, due to the interference between the back reflected light from the hemisphere surface and the light emitted into the mirror, a weak cavity effect. For the hemisphere with the cover glass substrate (not shown) the radiated light emitted through the top is 63%, and through the bottom, i.e. into the cover glass substrate, is 37%. These results show that the radiated power through the top interfaces only increases marginally when the DBR substrate is compared with the cover glass although emitted light is confined to a smaller numerical aperture for the DBR.

We then compare the super-sphere (with a radius of $4.16\,\upmu \hbox {m}$) to the hemisphere. The electric field magnitude of this super-sphere is shown in Fig. 4c and the far field projection in Fig. 4d for the $\mathrm {SiO_2/ Ta_2O_5}$ DBR substrate. Here we observe that the super-sphere acts as a lens to reduce the maximum collection angle, further limiting numerical aperture. The upward light emission is more strongly confined in a smaller half angle of approximately $8^{\circ }$, which relates to a numerical aperture of approximately 0.14 (on the DBR substrate). Considering all light emitted, that increases to a maximum numerical aperture of 0.34. Even for the cover glass substrate (not shown) most of the light is confined within a numerical aperture of approximately 0.5. The transmission through the entire top is approximately 68.5% and 31.5% into the DBR substrate. These values are similar to the ones presented for the hemisphere on the DBR substrate but confined to much smaller angles. Although the losses into the DBR substrate are reduced, a bigger difference between the super-sphere and the hemisphere is found for the cover glass substrate. In the case of the super-sphere on the cover glass approximately 55% is emitted through the entire top and approximately 45% into the cover glass substrate. The losses into the substrate are over 10% higher than for the DBR substrate as well as 7.5% higher compared to the hemisphere on the cover glass. Both structures also enhance the excitation of the dipole because of their respective lensing effects leading to higher focal intensities (Serrels et al. 2008b).

In comparison, for $\hbox {NV}^{-}$ centres coupled to hemispheres (Hadden et al. 2010) or bullseye gratings (Li et al. 2015) in bulk diamond, coupling efficiencies into the collected mode of up to 30% have been found. For nanodiamonds placed on the flat surface of zirconium dioxide hemisphere coupling efficiencies up to 18.3% are reported (Schröder et al. 2011). These coupling efficiencies highlight the clear advantage of the DBR structure which would more than double the coupling efficiency into the collected mode. Note, this coupling does not include further optical components in the system.

**Fig. 5:**
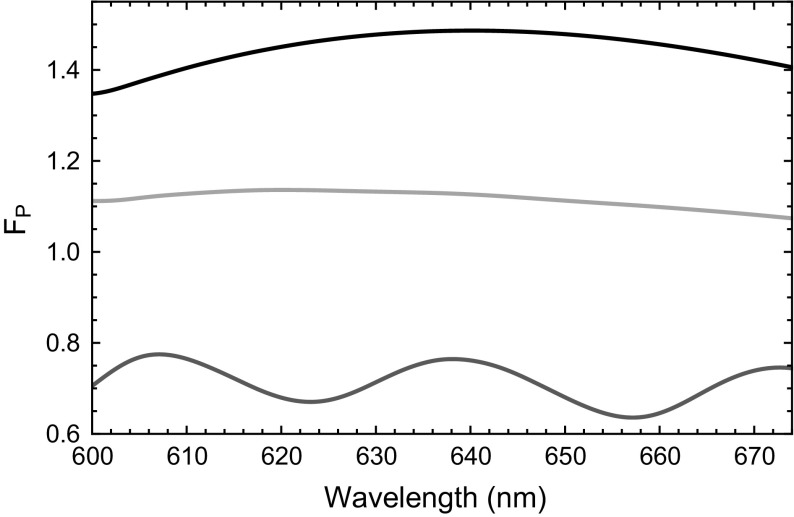
Simulated Purcell factors: *Blue* for the hemisphere shown in Fig. 4a without a top DBR; *Red* infinite polymer containing dipole on top of DBR substrate; *Black* dipole in vacuum on top of DBR substrate. In all cases the dipole is in a distance of 30 nm from the DBR substrate. (Color figure online)

Next, we investigate the effect of the polymer lenses on the dipole emission rate. Then also investigate the effect of enhancing the weak cavity effect seen with bare hemispheres by adding a top mirror. The dipole emission rate is proportional to the local density of states which can lead to emission enhancement (or suppression), depending on the dipole position within the structure as first proposed by Purcell for radio waves (Purcell 1946) and later adopted by the optics community analysing changes in spontaneous emission (Yablonovitch 1987; Lodahl et al. 2004). Here we calculate it by the standard method of taking the ratio of total power emitted (at a particular wavelength) to total power emitted when the emitter is embedded in homogeneous polymer. The modification of the photonic environment by the presence of the hemisphere is expected to lead to a change in the Purcell factor when compared with the same emitter on top of a mirror. For the latter case it is expected that ${\text{F}}_{\text{P}}\ge 1$ especially for some combinations of dipole orientation and emitter to surface distance (Barnes 1998; Badugu et al. 2013). However, when the hemisphere is considered (Fig. 4a), we observe a suppression of the spontaneous emission with ${\text{F}}_{\text{P}}\approx 0.7$ at 637 nm (Fig. 5, blue curve). Thus the Purcell factor is lower, compared to the emission rates for the single dipole on top of the DBR embedded in an infinite polymer (Fig. 5, red curve) and the dipole on top of the DBR in vacuum (Fig. 5, black curve). For these we find Purcell factors of 1.15 and 1.45 respectively.

**Fig. 6:**
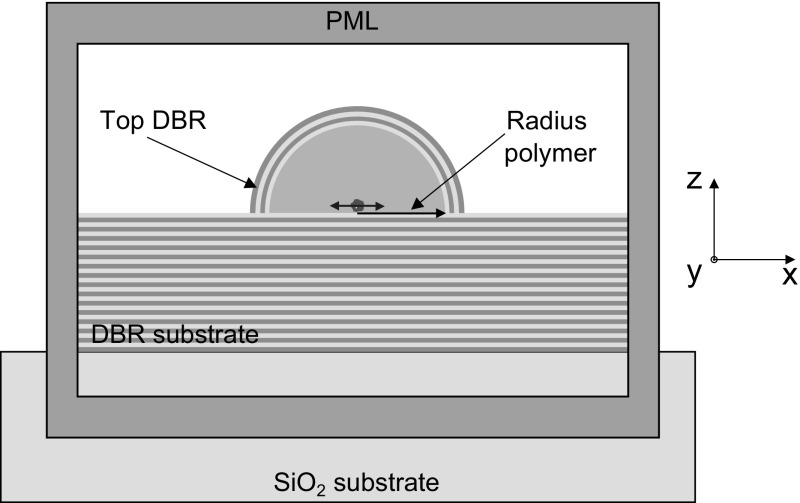
Simulation setup for hemispheres adding a top coated DBR

**Fig. 7:**
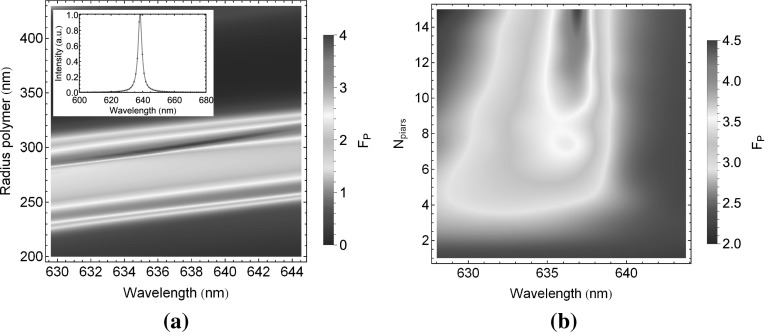
**a** Variation of polymer hemisphere radius with fixed 10 pair DBR (*inset* resonance spectrum at radius of 302 nm). **b** For a optimal radius at 302 nm modulated number of top DBR pairs

However, by coating hemispherical structures with DBR layers a stronger cavity effect is obtained leading to a Purcell enhancement of emission with respect to the unstructured environments. For the top DBR we utilise the same materials as for the substrate, illustrated in Fig. 6. We show false colour plots of the Purcell factor as a function of the inner radius of the polymer hemisphere and wavelength in Fig. 7a for a fixed number of 10 pair top DBR. We find for the fundamental mode at an optimal radius of 302 nm (at 637 nm) a Purcell factor of 4, corresponding to a cavity quality factor (Q-factor) of about 220. To understand future fabrication requirements we model at the optimal hemisphere radius of 302 nm the number of top DBR pairs (Fig. 7b). We find that even for a single DBR pair a Purcell factor of 2 can be achieved. This Purcell factor increases to 4.5 for a 15 top pair DBR. However, in practice Q-factors and Purcell factors will be limited by fabrication tolerances.

## Proposed experimental concept for the realisation of polymer structures

**Fig. 8:**
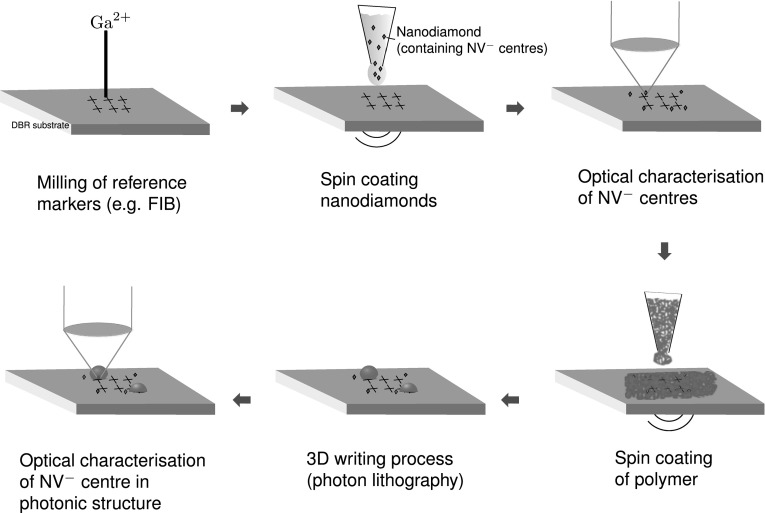
Experimental scheme for the realisation of nanodiamonds containing single nitrogen-vacancy centres addressed to polymer hemispheres and super-spheres

For the experimental realisation of these hemispheres and super-spheres, we propose the concept shown in Fig. 8. First, reference markers are milled into the surface of the DBR substrate, e.g. with a focused-ion beam (FIB). Afterwards, nanodiamonds containing $\hbox {NV}^{-}$ centres in solution are spin coated onto the substrate’s surface. Single $\hbox {NV}^{-}$ centres are then addressed relative to the reference markers and optically characterised. This characterisation would include the dipole orientation, optical power saturation, correlation function second-order of the single emitter. It follows the spin coating of photoresit, i.e. polymer, and the writing of the here presented hemispheres and super-spheres on top of precharacterised and localised $\hbox {NV}^{-}$ centres. Finally, the $\hbox {NV}^{-}$ centres coupled to these photonic structures need to be optically characterised again, in particular the coupling rate and angular orientation of the optical dipole.

## Conclusion

In this paper we presented modelling results for efficient coupling of nanodiamonds containing single colour centres to polymer structures on distributed Bragg reflectors. We explained how hemispherical and super-spherical structures confine light in small numerical apertures, emitted by such deterministically addressed colour centres. Coupling efficiencies of up to 65% within a numerical aperture of 0.75 for hemispherical and 68.5% within a numerical aperture of 0.34 for super-spherical structures are found. Further, we show how Purcell factors up to 4.5 with a total of 16 pairs of substrate and 15 top pairs distributed Bragg reflector can be achieved for wavelength scale hemispheres. With careful spectral and spetial mode engineering and colour centres with low phonon coupling, these structures may hold a key element to potential resonant scattering experiments (Noda et al. 2007; Androvitsaneas et al. 2016). We concluded with an experimental proposal for the realisation of the here simulated polymer structures.
